# Further records of non-cryptic New Zealand earthworms

**DOI:** 10.3897/zookeys.160.2354

**Published:** 2011-12-29

**Authors:** Robert Blakemore

**Affiliations:** 1 Department of Engineering Science, The University of Auckland (Te Whare Wānanga o Tāmaki Makaurau), New Zealand

**Keywords:** Eco-taxonomy, Annelida: Oligochaeta, new taxa, ICZN, island biodiversity

## Abstract

Current descriptions add natives *Aporodrilus aotea* **sp. n.**, *Aporodrilus ponga* **sp. n.** and *Notoscolex repanga* **sp. n.**, plus new exotic records to the numbers of megadrile earthworms known from New Zealand, which are now raised from 193 to 222 species in five families, *viz*: Acanthodrilidae, Octochaetidae and Megascolecidae, plus Lumbricidae and Glossoscolecidae for exotics. Overlooked spermathecal diverticula have been located for *Notoscolex equestris* Benham, 1942 and for *Megascolex animae* Lee, 1959 and non-tubular prostrates were misconstrued as tubular in *Megascolides tasmani* Lee, 1959. Of these latter three species, a lectotype is designated for *Notoscolex equestris* and holotypes of the other two are briefly redescribed. Whereas *Megascolides tasmani* now belongs in *Notoscolex* Fletcher, 1887 and *Megascolides animae* belongs in *Anisochaeta* Beddard, 1890, further lack of dorsal pores in *Notoscolex equestris* as with *Notoscolex esculentus* (Benham, 1904) and *Notoscolex mortenseni* (Michaelsen, 1924) newly qualifies all three as additional combs. novae in primarily Tasmanian genus *Aporodrilus* Blakemore, 2000.

## Introduction

The definitive earthworm study completed 50 years earlier (Lee 1959) was not taxonomically reviewed until Dr Ken Lee (1927-2007), my PhD assessor and mentor, kindly invited me to compile an update in 1999 for a *NZ Species 2000* meeting to be held around January, 2000 at Te Papa Museum in Wellington, New Zealand (NZ). This was, however, not finally published until 10 years later as modified under Glasby et al. (2009). Whereas the seminal works by Lee (1952, 1959) culminated in approximately 193 species in two families, subsequent lists by Blakemore (2000a, 2004, 2006, 2010) totaled 214 taxa in five families with some names removed and several others added.

Smith (1886) had earlier remarked that “*The habits of New Zealand earth-worms receive the smallest share of attention from naturalists of any group of our native fauna. This is to be expected, as the study of worms requires much time and patience*”. Similarly, little attention has been shown to the native earthworms following Lee’s detailed studies 50 yrs before. For exotics, the main additions to Lee (1959) were of *Aporrectodea tuberculata* (Eisen, 1874) and *Octolasion lacteum* (Örley, 1881) by Martin (1977), and three additions to the NZ alien species list following extensive searches of literature by the current author (e.g. Blakemore 2002, 2004, 2006, 2008). These were of records from NZ by Michaelsen (1900: 425, 1903: 132) of *Pontoscolex corethrurus* (Müller, 1857) that were seemingly overlooked by subsequent researchers; by Easton (1981: 53, 1984: 118) of *Amynthas hupeiensis* (Michaelsen, 1895) plus *Amynthas gracilis* (Kinberg, 1867) and *Amynthas corticis* (Kinberg, 1867) from Raoul Island. The latter was already described as widespread by Lee (1959) but under the synonymous names of “*Pheretima peregrina* (Fletcher)”, *Pheretima clerica* Benham, 1947 and *Pheretima campestris* Lee, 1952. Additionally, *Perionyx excavatus* Perrier, 1872 and *Dendrobaena veneta* (Rosa, 1886) were identified by the current author around 2001 from vermicomposting operations in NZ (Blakemore 2002). Other new records of four or five other exotic species are pending (Blakemore submitted).

For natives, few had subsequent reports and because of this approximately 77 were automatically listed as “Threatened” or “Endangered” in the Department of Conservation (DoC) threatened species list (Anon. 2005). Details on three of these are given in McGuinness (2001) while 168 species from 173 qualified as “data deficient” in Hitchmough (2002) and Hitchmough et al.(2007). Terrestrial surveys continue to inexplicably languish. Further work such as that conducted by Springett and Gray (1998) is urgently required to determine the true status and ecology of natives and the extent of the relatively few species of introduced lumbricids and other exotics.

A study by Blakemore (2010) added twelve natives plus a new New Zealand record of *Octolasion tyrtaeum tyrtaeum* (Savigny, 1926), and synonymized genus *Eudinodriloides* Lee, 1959 with *Decachaetus* Lee, 1959. A taxonomic checklist gave natives separate family status to raise the numbers of megadrile earthworm families known from New Zealand from three to five, *viz*. Acanthodrilidae, Octochaetidae and Megascolecidae *sensu* Blakemore (2000c), plus exotic Glossoscolecidae (for *Pontoscolex*) and Lumbricidae, with species then totaling 214. In contrast, some contemporary online and public presentations (e.g. http://soilbugs.massey.ac.nz/oligochaeta.php, http://www.terranature.org/weta.htm, http://www.teara.govt.nz/en/1966/worms-earth/1, www.doc.govt.nz/upload/documents/science-and.../casn320a.pdf) yet claim just ~173 native species plus ~20 exotics in only two or three families.

Buckley et al. (2011) recently posited a phylogeny for the New Zealand earthworm fauna under (Oligochaeta: Megascolecidae) which they comprised as “Megascolecinae and Acanthodrilinae” based on approximately 33 newly collected known natives and about 48 unknown cryptic natives (total 81 taxa). However, their concepts of families and genera appear to be more than 50 yrs old reversions and, as no species/genera types were sourced, confidence in taxonomic acuity at even species level is reduced and their phylogenetic conclusions may be questionable. For instance, Buckley et al.(2011: 86) mention “a review of acanthodriles from Tasmania” when there are none. Moreover, their suggestions of cryptic taxonomic diversity in their 48 unknowns without consideration and analysis of types of all synonyms under ICZN Principals of Priority and of Typification may also be premature (see Blakemore et al. 2010). Speculations in Buckley et al. (2011), in particular a lengthy repetition of the merits of non-New Zealand *Terrisswalkerius* Jamieson, 1994 (they consistently misspell “*Terriswalkerius*”), were supported neither with analyses of type-specimens of genera nor of type-species of senior synonyms. This and other oversights are discussed in a summary endnote to this paper.

Changes invoked by Blakemore (2000a, 2000b, 2000c, 2004, 2006, 2008, 2010), and currently, compared to Lee’s (1959) original, and clarifying some reversions/omission or errors in Buckley et al. (2011) concerning native species/genera are:

• Restatement of validity of Acanthodrilidae, Octochaetidae and Megascolecidae (plus Exxidae at one time thought from NZ) as separate families.
• Neoendemic *Microscolex macquariensis* (Beddard, 1896) is removed as Macquarie Island is now claimed by Australia (see Blakemore 2006), albeit still cited by Buckley et al. (2011) as a native but misspelt as “*Microscolex maquariensis*”.
• Because *Rhododrilus disparatus* Lee is meroic it was transferred as a new combination in *Leucodrilus* Lee by Blakemore (2004, 2010).
• *Octochaetus* was proven to have native Australian representatives too, e.g. the native *Octochaetus ambrosensis* (Blakemore, 1997) and similar species in Queensland where *Adroitplema* Blakemore, 2006 (nom. n. pro *Neodiplotrema* Dyne, 1997 non Yamaguchi, 1938) is now a junior synonym (see Blakemore 2000c, 2006). [Cf. miscitation of the genus by Buckley et al. (2011)].
• *Sylvodrilus* Lee is retained as the type is anisochaetine, i.e., classed as non-lumbricine (cf. *Eudinodriloides*).
• *Plutellus* Perrier species are transferred to *Graliophilus* Jamieson which is said to have tubular prostates (as “flattened tubes”) in its type species; those species having non-tubular prostates more appropriately belong in *Zacharius* Blakemore, 1997.
• *Megascolides* McCoy, 1878 is retained, although species with non-tubular prostates are returned or reallocated to *Notoscolex* Fletcher, 1886/7 for which its junior synonyms are: *Tokea* Benham, 1904; ?*Nelloscolex* Gates, 1939; ?*Lennoscolex* Gates, 1960; *Pseudonotoscolex* Jamieson, 1971; *Pseudocryptodrilus* : Jamieson, 1974, 2000 (part. cf. *Megascolides*); *Oreoscolex* Jamieson, 1973; *Araucaridrilus*, Jamieson, 2000;?*Plutelloides* Jamieson, 2000 (but cf. *Megascolides*) – synonyms from Blakemore (2000c, 2005a, 2006, 2010). *Megascolides* is a classical genus lacking nephridial bladders, cf. classical *Cryptodrilus* that retains and/or obtains them.
• Endemic *Perionyx* Perrier, 1872 spp go into originally defined *Perionychella* Michaelsen, 1907 [syn. *Terrisswalkerius* – for its putative type *Perichaeta canaliculata* Fletcher, 1887 and similar species with non-tubular prostates – see Blakemore (2000c) and cf. those species misplaced under that genus name that actually have tubular prostates and thus belong in *Diporochaeta* or *Reflechtodrilus*].
• *Diporochaeta* Beddard, 1890 is retained with its original definition [including the balance of *Terrisswalkerius* spp (part. – but not type or other species with non-tubular prostates – see Blakemore 2000c and cf. *Perionychella*), some other erstwhile *Terrisswalkerius* interlopers properly belong in *Reflectodrilus* Blakemore, 2005 as per Blakemore (2005a, 2006)].
• *Perionychella shoeana* (Cognetti, 1912) position is rendered uncertain by its original description as: “Each prostate is a tongue-shaped body, not divided into lobes” being revised by Lee’s (1959: 325) inspection of new material (the type not being located) to “Prostates short tongue-shaped organs, projecting laterally through xviii [18], surrounded by thin sheath and each consisting of a number of distinct lobes”. Nevertheless, having non-tubular prostates qualify it for *Perionychella*; cf. Buckley et al.’s (2011: Appendix) inappropriate naming as “*Perionyx shoeanus*” (sic).
• *Megascolex* Templeton, 1844 species from Australia and New Zealand are now placed in *Anisochaeta* Beddard, 1890 for which *Trichaeta* Spencer, *Spenceriella* Michaelsen, *Gemascolex* Edmonds & Jamieson, *Pericryptodrilus* and *Propheretima* Jamieson are junior synonyms (see Blakemore 1997, 2000b, 2000c, 2002, 2005a, 2006, 2010).
• Species having tubular prostates and previously placed in *Spenceriella* (the neotype of which was stated to have racemose prostates, although this is possibly a mistake - see Blakemore 1997: 1823; 2000b, c) are now in the next available genus, *Celeriella* Gates, 1958 for which *Pericryptodrilus* Jamieson, 1977 would be a synonym if the prostates are indeed “thickly or flattened tubular” as claimed (but as they appear tubuloracemose then this name likely belongs in *Anisochaeta*). *Celeriella* is primarily an Indian genus and it is probable that its New Zealand species will eventually go into a separate genus (as noted by Blakemore 2006).
• Monotypic *Eudinodriloides* Lee, 1959 was placed under *Decachaetus* Lee, 1959 in Blakemore (2010) with its perichaetine type-species, *Decachaetus forsteri* (Lee, 1959), comb. n.; cf. Buckley et al. (2011: Appendix A) with a single “*Eudinodriloides* n. sp. 2” (sic).
• Blakemore (2005a) noted that Lee (1962) made *Spenceriella shakespeari* (Benham) junior synonym of *Megascolex antarcticus* Baird, itself transferred to *Celeriella* Gates although this appears to have been overlooked or ignored by Buckley et al. (2011: Appendix A) who yet claim to have sampled “*Spenceriella shakespeari*” (WM93 from Waharau Pk, Hunua Range) but appear not to have sequenced its DNA.
• All “Michaelesen (1923)” species should be changed to Michaelsen (1924) according to the volume preface (see http://ia700402.us.archive.org/4/items/videnskabeligeme74dans/videnskabeligeme74dans_bw.pdf accessed Sept., 2011) and one of these, *Notoscolex mortenseni*, is considered herein.

## Methods

Specimens were sketched, dissected and described under low power microscope using the techniques and conventions noted in Blakemore (2000, 2002, 2008). Tissue samples were taken from new type-species to attempt DNA/COI barcode analysis – any results are to be posted in GenBank. Classification follows Blakemore (2000) at family level and Blakemore (2002, 2008, 2010, 2000b) at genus and species levels. Discussion is confined to comments after species descriptions and endnote summaries of taxonomic conclusions.

## Results

### New species descriptions

#### Aporodrilus aotea sp. n.

urn:lsid:zoobank.org:act:F014AE5E-D434-4119-B949-E5AB86DB1E4E

http://species-id.net/wiki/Aporodrilus_aotea

Fig. 1

##### Material Examined.

Holotype Auckland Museum; AMNZ 5254. Single complete specimen, now dissected, from New Zealand, Great Barrier Island, Little Windy Hill (ca. 36°10'S, 175°23'E). Coll: 2.IX.2001, J.W. Early & R.F. Gilbert. “*Under rock on forest floor. L11002*”. “*W-025*” on lid. (Small tissue sample was taken for DNA analysis - code RJB09).

**Etymology.** After Maori name for Great Barrier Island; *Aporodrilus* is treated as masculine but this place name remains genderless as a noun in apposition.

**Diagnosis.** *Aporodrilus* having spermathecal pores paired segmentally in 6, 7 and 8; holandry with seminal vesicles in 9 and 12; oesophageal glands annular in 10–14; large genital markings paired in 17/18 and 18/19 on either side of male pores.

##### External characters.

Body circular tapering at both ends. Dark, matt grayish pigment with iridescent cuticular sheen; paler intersegments and setal auriolae. Length 140 mm with 75 segments. Prostomium epilobous. Setae lumbricine, 8 per segment in rows becoming increasingly irregular further back. Clitellum not well marked. Dorsal pores absent. Nephropores not found (meroic). Spermathecal pores segmental, equatorial just below setae a on 6, 7 and 8. Female pores mid-ventral pair anteriormedian to setae a on 14. Male and prostatic pores combined on tiny mounds on 18 in position of deleted setae a. Penial setae not found. Genital markings large, longitudinally symmetrical pads, paired in 17/18 and smaller in 18/19.

**Figure 1.**
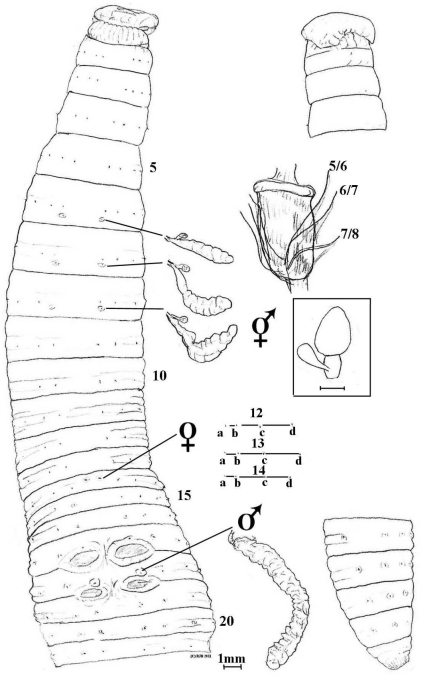
*Aporodrilus aotea* sp. n. ventral view with dorsal view of epilobous prostomium, spermathecae, prostate and gizzard in 5 *in situ*; and lumbricine setal ratios on 12–14; plus lateral view of tail end. [Boxed spermatheca is for comparison of *Aporodrilus esculentus* (Benham, 1904) from Benham’s fig. 67 and from Lee (1959: fig. 309)].

##### Internal morphology.

Pharyngeal mass to 4. Septa 4/5-10/11 thin, only 11/12/13 with slight thickening and thereafter membranous. Gizzard strong and elongate apparently in 6-7 but discernable in 5 by tracing septum 5/6 to near base despite dorsal-wards displacement. Dorsal blood vessel single; hearts paired and increasingly large in 9-13; supra-oesophageal vessel in 10-13. Nephridia meroic with forests of avesiculate tubules on body wall. Spermathecae in 7, 8 and 9 each with elongate, flaccid ampulla and single, small, clavate diverticulum (inseminated) near base implicated with anterior septum which is transgressed. Holandric: minute funnels in 10 and 11 ventrally; seminal vesicles paired, racemose posteriorly in 9 and anteriorly in 12. Ovaries paired as free egg-string bunches ventrally in 13; ovisacs not found. Prostates tubuloracemose extending to ca. 22 from small flaccid ducts to male pores in 18. Oesophagus with oesophageal glands small in 10 and larger in 11-13 then small again in 14; glands more saccular than composite but dilated compared to extraneous oesophageal width. Intestinal origin in 16. Typhlosole and caeca not found (absent). Gut contains fine colloidal reddish soil.

##### Ecology.

Lack of dorsal pores is usually associated with aquatic habitat, but possibly also with high rainfall/soil-moisture, however, the strong gizzard suggests a loamy diet. Further ecological and/or behavioural information is wanting.

##### Remarks.

*Aporodrilus aotea* compares with *Aporodrilus mortenseni* (Michaelsen, 1924) that differs, not least, by having its three pairs of spermathecal pores intersegmental in 6/7/8/9 and by lacking genital markings. However, in the review by Lee (1959) that did not routinely note presence or absence of dorsal pores (nor genital markings), the current species keys out nearest to Lee’s *Megascolides* species now in *Notoscolex*, viz.: *Notoscolex sapidus* that differs in its spermatheal pores intersegmental in 6/7/8/9; or to those now in *Aporodrilus* viz. *Aporodrilus equestris* (Benham, 1942), and edible *Aporodrilus esculentus* (Benham, 1904) with which it perhaps comes closest as this too has spermathecae opening on 6-8. *Aporodrilus equestris* as redescribed below has genital markings elongate in 17 & 19, exceptionally thickened septa, a gizzard in 6 and intestine from 17; while *Aporodrilus esculentus* has genital markings paired midventrally in 16 and 17, thicker septa, a smaller gizzard, oesophageal dilations only in 15 and its spermathecae of a more spherical and compact form (see figures and compare Benham’s original sketches http://www.archive.org/stream/proceedingsofzoo19042zool#page/240/mode/2up). A more distant contender is *Notoscolex urewerae* (Benham, 1904) “a short white worm” that has genital marking mid-ventrally in 19/20 and last hearts in 12 amongst other differences (its dorsal pores are unrecorded and possibly it too belongs in *Aporodrilus*).

#### Aporodrilus ponga sp. n.

urn:lsid:zoobank.org:act:C7CD2EDF-0B2A-4EEA-A34B-4CF06F927A04

http://species-id.net/wiki/Aporodrilus_ponga

Fig. 2a 2b

##### Material Examined.

Holotype Auckland Museum; AMNZ 5255. Single mature, posterior amputee rather poorly preserved from Waitakere Ranges, Waiatarua. Coll: 9.V.1995, G. Ripley. “*Nikau/Ponga forest L761*”; “W-012” on lid. (Small tissue sample was taken for DNA analysis coded RJB10). [Two other specimens from the same jar are a posterior portion of a worm (AMNZ 5256) matching the dimensions and frayed edge of the current specimen is itself missing its tip; the other (AMNZ 5254) is a large mature, anterior amputee that is certainly different and probably a new species but which is inadequate for formal description here].

##### Etymology.

After Maori name for silver fern *Cyathea dealbata* (G. Forster) Swartz, 1801, from the habitat detail and also the symbol commemorating the All Blacks victory in 2011 Rugby World Cup; *Aporodrilus* is masculine, but a noun in apposition is genderless.

##### Diagnosis.

*Aporodrilus* having spermathecal pores paired intersegmentally in 7/8/9; metandric with seminal vesicles in 12; no oesophageal glands; genital marking as a distinct pad in 17/18 with male pores on lower rim replacing setae a.

##### External characters.

Body robust, dorsally canaliculated in parts before amputation. Pale putty coloured in alcohol. Length 220+ mm anterior portion (a posterior fragment in jar is also 220mm and if from same specimen would give length = 440 mm). Prostomium much wrinkled prolobous. Setae lumbricine, obscure in anterior and mostly occluded on clitellum apparently converging towards male pores; further back the rows except for setal a lines become progressively irregular. Clitellum slightly more tumid and yellowy in ½13–17 (or thereabouts). Dorsal pores absent. Nephropores absent (meroic). Spermathecal pores intersegmental, detected by probe from interior and approximately in setal a lines in 7/8/9. Female pores large paired on 14 (setae obscure) in line with setae a of 13. Male pores superficial on 18 in place of deleted setae a on bottom rim of pad (detected by probe internally). Penial setae not found. Genital marking as a large pad in 17/18 distending both adjacent segments.

**Figure 2a.**
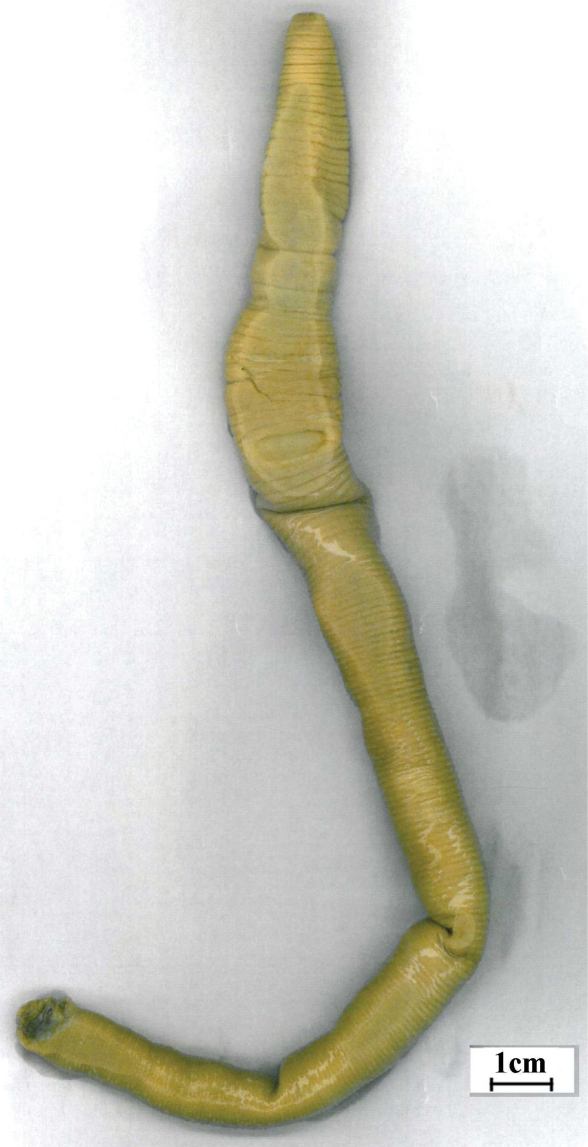
*Aporodrilus ponga* ventral scan of Holotype (colour).

**Figure 2b.**
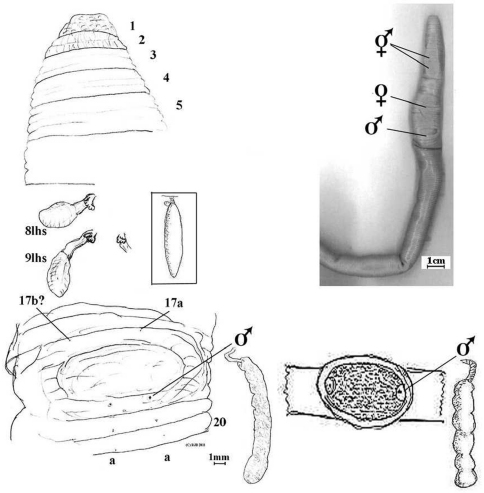
*Aporodrilus ponga* dorsal view of prolobous prostomium, spermathecae (8lhs and 9lhs and part of 9rhs) and prostate in 18lhs *in situ*. Male field is shown with setae 17b? and 17a marked (setae a occluded by male pores on 18). [Boxed spermatheca of *Notoscolex hakeaphilus* Benham, 1949, with Benham’s sketch of its male field and prostate shown for comparison].

##### Internal morphology.

Septa and pharyngeal mass absent before 5, septa 5/6–12/13 greatly thickened, thereafter membranous. Gizzard mucular barrel in 5. Dorsal blood vessel single; hearts sinuous in 9–13. Nephridia meroic forests on body wall. Spermathecae paired in 8 and 9 each with flask-shaped ampulla on equally long flat duct with multilocular diverticular frill (inseminated) near base. Probably metandric as paired seminal vesicle seen in 12 only. Testis and ovaries not located, probably minute and lost in musculature of septa and body wall. Prostates rounded but finely incised throughout so not as found in Acanthodrilidae and Octochaetidae (cf. Exxidae), i.e. tubuloracemose with small flaccid ducts in 18. Oesophagus without noticeable dilations (what I initially took as a hemispherical thickening of posterior of 9 was determined as a septum). Intestine substantial yet dilated and easily ruptured, origin appears in 15 or 16. Gut contains finely ground organic matter, organic soil plus coarse multi-coloured grits.

##### Ecology.

Anterior musculature and thickened septa are associated with strong burrowing, and lack of (anterior) dorsal pores may aid maintenance of hydroskeletal turgor pressure.

##### Remarks.

*Aporodrilus ponga* differs from *Aporodrilus aotea* on almost each specific point. According to Lee (1959), who often took tubuloracemose prostates to be tubular, this specimen keys to genus *Megascolides* but fails to match any known taxa from there. If more properly allowed into Lee’s *Notoscolex* the similarity with *Notoscolex hakeaphilus* Benham, 1949 is remarkable: viz. large size (650–950 mm) with irregular setae, male pores on a median oval depression, septa absent before 5, spermathecae in 8 and 9, and metandry. Presumed differences however, are darker colour (current specimen bleached in alcohol?), epiloby and again much furrowed as here, tufted nephridia (how tufted?) in anterior, a thick-walled enlargement of oesophagus in 8 (possibly as I initially thought was in 9), prostates claimed as flat rather than rounded (although figured as rounded), spermathecae with “*a minute globular diverticulum*” (variation?), and male pores shown laterally within pad on 18 rather than on its rim as here. It is possible Benham mistook some of these points. His report of last hearts in segment 10 for this species is undoubtedly anomalous as invariably they are in either segments 12 or 13 in normal Megascolecidae; and intestine in 12 is also anterior to what is usual. No mention was made of dorsal pores by him. Benham’s type was collected in 1946 from Kerikeri (A.48.31 – supposedly in poor condition but confirmation from Otago museum unforthcoming) that he thought imported from Australia as was the plant it was found under. This seems unlikely for such a large species: even if its cocoons were introduced, large species often have particular habitats unlike most small to medium cosmopolitans. Lee (1959: 318), presumably accepting Benham’s characterization, has another specimen (current location unknown) from Pukehohe, suburb of Auckland, from subsoil collected by W. Cottier in 1951. (An online GBIF record of Australian Museum AM W.29352 at Taupo is unconfirmed http://data.gbif.org/occurrences/237279142 accessed November, 2011). A much smaller species but with remarkable superficial similarity of marking to Benham’s *Notoscolex hakeaphilus* is his *Notoscolex maoricus* (Benham, 1904) (syn. *Tokea decipiens* Benham, 1905) that also comes from “*Waitakerei Bush*” (= Waitakere), near Auckland.

Without information to the contrary we must reluctantly accept the balance of Benham’s earlier diagnosis, in which case a new name for this specimen has merit. Confirmation of independence of either species now depends on reinspection of Benham’s type, apparently beyond the brief, budget and resources of successive workers for the last 62 years, including the present one.

#### Notoscolex repanga sp. n.

urn:lsid:zoobank.org:act:796E44B7-47A8-4D10-B491-8BBA8B6A85C3

http://species-id.net/wiki/Notoscolex_repanga

Fig. 3

##### Material Examined.

Holotype Auckland Museum; AMNZ 5253. Single complete specimen, now dissected, from New Zealand, Cuvier Island (36°26'S, 175°46'E) SE catchment 40–60 m. Coll: 3.IV.2000, J.W. Early & R.F. Gilbert. “*Under rock in stream bed. L8229*” “*W-024*” on lid. (Small tissue sample was taken for DNA analysis coded RJB07).

##### Etymology.

After Maori name for Cuvier Island; *Notoscolex* is treated as masculine but this place name remains genderless as a noun in apposition.

##### Diagnosis.

*Notoscolex* having spermathecal pores paired posteriorly in 7 and 8 but with clavate spermathecae anteriorly in 8 and 9; holandry with seminal vesicles in 9 and 12; oesophageal gland annular in 12; genital markings mid-ventral in 16/17/18/19.

##### External characters.

Body circular. Pale unpigmented in alcohol. Length 135 mm with 149 segments. Prostomium prolobous. Setae lumbricine, 8 per segment in mostly regular rows and almost equidistant throughout. Clitellum not marked. Dorsal pores present but minute and difficult to detect, possibly commencing from 9/10 or 10/11. Nephropores not found (meroic). Spermathecal pores segmental, posteriorly just above intersegments in setal a lines on 7 and 8. Female pores difficult to detect with certainty, possibly mid-ventral pair anterio-median to setae a on 14. Male and prostatic pores combined on small tumescences on 18 in position of deleted setae a. Penial setae not found. Genital markings mid-ventral eye-shaped sucker pads in 16/17, 17/18 and 18/19; a yellowy midventral patch from ½14-16/17 may be artefactual.

**Figure 3.**
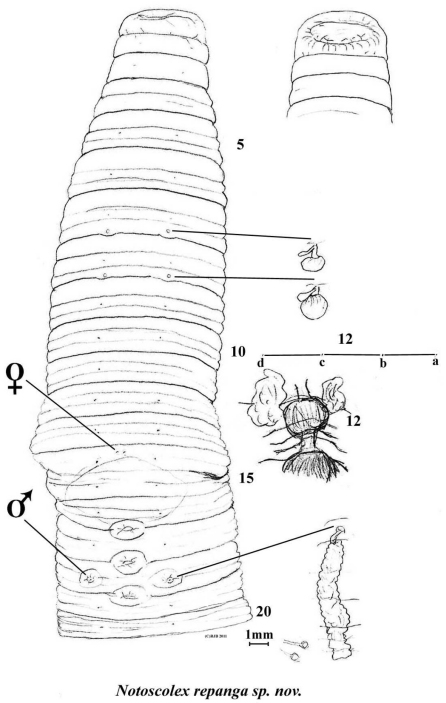
*Notoscolex repanga* ventral view with dorsal view of prolobous prostomium, spermathecae, prostate and oesophageal gland in 12 *in situ*; and lumbricine setal ratio in 12. (Small structures near scale bar are probable unidentified parasites, attached on intestine in region of 35–40).

##### Internal morphology.

Septa increasingly thickening from 4/5–10/11; 11/12 thin and thereafter membranous. Gizzard large but weak in 5. Dorsal blood vessel single; commissurals in 5–8; hearts paired and small in 9, much larger in 10–13; supra-oesophageal vessel not found. Nephridia meroic with several avesiculate tubules almost evenly spaced in several rows on body wall in each segment. Spermathecae in 8 and 9 each with saccular ampulla and single, small clavate diverticulum (non inseminated). Holandric: testes and funnels minute in 10 and 11 ventrally; seminal vesicles paired, racemose in 9 and, larger, in 12. Ovaries paired as fine string masses ventrally in 13; ovisacs not found. Prostates flattened, tubuloracemose extending to ca. 24 from small ducts to male pores in 18. Sessile tumidity associated with genital markings internally. Oesophagus large and folded in on itself in anterior in 6–9 at least; with annular dilated oesophageal gland in 12. Intestinal origin in 16. Typhlosole and caeca not found (absent). Gut contains fine silt with few organic fragments. Intestine paler and concertinaed between 35–40 where several (gregarine?) parasitic cysts on stalks attach to it (see figure).

##### Ecology.

Habitat location (under rocks in stream) would indicate an aquatic or semi-aquatic life style, a conclusion supported by the pale colouration plus reduced dorsal pores and gizzard; while the folded oesophagus in the anterior would allow considerable extension (for movement and feeding) and the gut contains silty (alluvial?) soil. Alternatively, this specimen may be an unintentional interloper washed into the stream from adjacent soil; more ecological information is needed to confirm or disconfirm this.

##### Remarks.

*Notoscolex* is primarily an Australian genus with representatives in Sri Lanka and southern India as well as NZ. The current specimen although large is possibly subadult (it has genital markings but lacks a distinct clitellum and spermathecae uninseminated) yet appears to be a distinct species. Its morphology is comparable to the nine previously known regionally compatriot *Notoscolex* species, all confined to the north of the North Island, many of which were at some time placed in the cohesive genus *Tokea* Benham, 1904. This latter genus was made junior synonym of *Notoscolex* following Michaelsen (1916), Stephenson (1930: 837) and as herein compared to Lee (1959) who placed it within *Megascolides*.

Of the nine or so New Zealand species now known, *Notoscolex repanga* differs: from *Notoscolex sapidus*, *Notoscolex urewerae*, *Notoscolex huttoni* and *Notoscolex suteri*, all by Benham (1904) and each having three pairs of spermathecae; and from Benham’s *Notoscolex kirki* and *Notoscolex maoricus* which share two pairs but differ in their arrangements of genital markings and spermathecae. Whereas *Notoscolex kirki* has intersegmental spermathecal pores in 7/8/9, *Notoscolex maoricus* has them segmentally but in posterior of 7 and 8, and not 8 and 9 as he originally stated and as inadvertently retained by Lee (1959: 302). [This correction according to Benham (1905: 240, pl XL, figs. 1–2, 8–9) – see http://www.archive.org/stream/transactionsproc38newz#page/240/mode/2up or http://rsnz.natlib.govt.nz/volume/rsnz_38/rsnz_38_00_002970.pdf where he unconventionally records segments 7 & 8 as “7/8” and 8 & 9 as “8/9”; see also http://rsnz.natlib.govt.nz/image/rsnz_38/rsnz_38_00_0736_0000f_ac_01.html for his original figures of *Tokea maorica* and its junior synonym *Tokea decipiens* Benham, 1905]. *Notoscolex hakeaphilus* (Benham, 1949) does have spermathecae in 8 and 9 (with pore locality indeterminate) but differs not least in its large (650 mm) dark body with irregular setae and metandry. *Notoscolex napierensis* (Benham, 1941) has its four pairs of spermathecal pores equatorial on 6–9, and was probably misdescribed by Benham as having two pairs of tubular prostates on 17 and 19, where the genital markings lie, while Lee (1959: 304), who thought it introduced, accords it a single pair of lobate prostates in 18; dorsal pores were unstated by both authors. A previous *Notoscolex* member, *Notoscolex mortenseni* (Michaelsen, 1924) is now moved to *Aporodrilus*.

Superficially, *Notoscolex repanga* is somewhat similar to several *Megascolides* spp., such as *Megascolides viridis*, *Megascolides raglani*, *Megascolides irregularis*, *Megascolides alba* and *Megascolides novaezealandiae*, but it differs from all generically by its non-tubular prostates, and specifically by virtue of combination of segmental spermathecal pores and three mid-ventral genital markings, plus an oesophageal gland in 12 only and its last hearts in 13 rather than 12.

A further *Megascolides* species occurring in NZ and now possibly extinct was described by Schmarda in 1861 under the title of “*Hypogaeon orthostichon*,” that is subjected to separate treatment in a forthcoming publication (Blakemore submitted.).

### Redescription of original AMNZtypes

Types are redescribed for *Anisochaeta animae* (Lee, 1959) and *Notoscolex tasmani* (Lee, 1959) comb. n., and newly designated for *Aporodrilus equestris* (Benham, 1942) comb. n. As with Tasmanian *Notoscolex tasmanianus* Fletcher, 1887, the erstwhile representative of temporary genus *Pinguidrilus* Jamieson, 1974, that was found by Blakemore (2000c) to have had its spermathecal diverticula overlooked, a similar oversight applies to types for both *Notoscolex equestris* Benham, 1942 and *Megascolex animae* Lee, 1959. While *Megascolides animae*,which belongs in *Anisochaeta*,has dorsal pores (from 4/5, pers. obs. – see separate description), *Notoscolex equestris* is found to lack them and thus, along with *Notoscolex esculetus* and *Notoscolex mortenseni*, belongs as comb. n. in *Aporodrilus* Blakemore, 2000.

#### Anisochaeta animae

(Lee, 1959)

http://species-id.net/wiki/Anisochaeta_animae

Fig. 4

Megascolex animae Lee, 1959: 281, figs. 301-304.
Anisochaeta animae ; Blakemore, 2004, 2005, 2010.

##### Distribution.

From Unuwhao Mt., nr Spirits Bay, northern extremity of Northland, NZ.

##### Description from type.

AMNZ 5038 for *Megascolex animae* has the following label in jar: “*Unuwhao nr. Spirits Bay, N.Z. Coll: A.W.B.P. Feb 1946 AM8 1039*”. It is a substantial specimen – 196 mm × 13.5 mm – collected by former director of Auckland Museum, A.W.B. Powell in Feb. 1946. Although the specimen in alcohol is now bleached of its earlier dark brown dorsal pigmentation and is wrinkled and hardened, it is yet well preserved. Reinspection of the type largely conforms to Lee’s original except that his figured spermathecae (from 9rhs that he included in a separate vial) is broken off after the diverticulum, and diverticula are newly found to be present in the remaining *in situ* spermathecae (see figure). The peristomium was not obviously cleft ventrally and genital markings, rarely described by Lee, were not found, but dorsal pores were: small in 4/5 and more obvious afterwards. Specimen AMNZ 5038 is the monotypic holotype fixed by original designation under (ICZN, 1999: Art. 73.1.1); the only slight ambiguity is that Lee (1959: 282) also noted another specimen with “*Same data as type material*” that has not been subsequently located.

**Figure 4.**
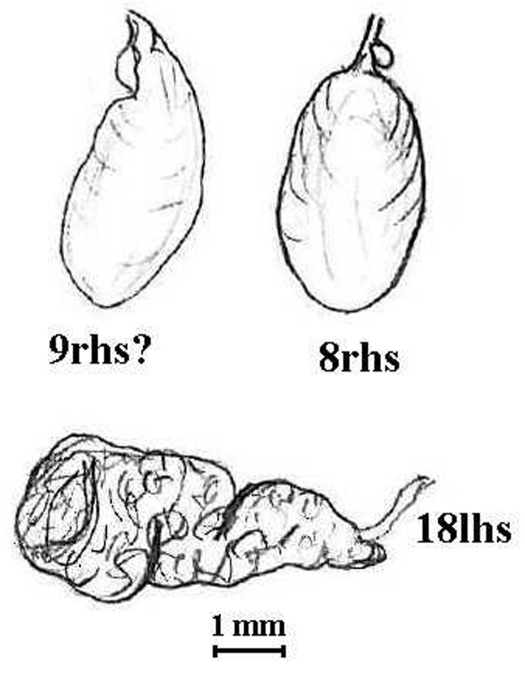
*Anisochaeta animae* (Lee, 1959). Holotype (AMNZ 5038); spermatheca labelled 9rhs? is from separate vial and is a broken off ampulla; that labelled 8rhs is *in situ* showing small iridescent diverticulum (no parasites were found internally); the tubuloracemose prostate is from 18lhs.

#### Notoscolex tasmani

(Lee, 1959)

http://species-id.net/wiki/Notoscolex_tasmani

Fig. 5

Megascolides tasmani Lee, 1959: 313, figs. 326-328.
Megascolides tasmani ; Blakemore, 2004, 2005, 2010; Buckley et al., 2011?

##### Distribution.

Known only from Great Island, Three Kings Islands. Buckley et al. (2011) claim to have “*Megascolides tasmani*” (WM82), but this must be a mistake if they could not identify it as clearly belonging in *Notoscolex* with non-tubular prostates as herein from type inspection (or perhaps they failed to attempt specimen dissections?).

##### Description from type.

Specimen AMNZ 5039 has the following labels in its jar: “*Auckland Museum Coll No. 13 Wet Greywacke gravel. Tasman Stm. Great Island. Three Kings 31.xii.52 J.S. Edwards*”; “*HOLOTYPE Megascolides tasmani Lee Tasman Stm, Great Is. Col. J.S. Edwards 30/12/52 1021. 3KI3*” [note slightly different dates], a further label is blank. Somewhere are two other non-type specimens from the same locality according to Lee’s account (Lee 1959: 314) with one collected “5.I.53”.

The type specimen is dark and brittle, shrunken to 53 mm long. Lee has 67.5 mm with 132 segments but it is a posterior amputee so must naturally be greater (there is also a slight possibility it acquires more setae posteriorly). Dorsal pores, not noted by Lee, are present from 10/11, at least. It appears lumbricine with widely spread setae that converge slightly towards male pores. Lee has overlooked the penial setae which are protruding (due to shrinkage?) from each of the male pores on 18. In 17 in ab lines is a reddish patch that may be a residual genital artefact. The female and spermathecal pores are no longer obvious. Vascularization is mostly as described by Lee, i.e., commissurals are in 6–9, hearts are in 10–12 from dorsal blood vessel that loops between septa in 11, 12, (not 13) 14, 15 and 16 thereafter single (Lee 1959: fig. 327 shows only in 14 and 15). Gizzard appears in 6 rather than 5 but being overlain by tufted pharyngeal glands it is difficult to discern. Seminal vesicles are in 9 and 12 as described. Prostates differ significantly as they are flattened tubuloracemose structures, rather than “tubular, convoluted” as Lee has them; the duct is not traceable in the delicate specimen and, moreover, there is a bundle of long reddish penial setae more ventrally. An excised spermatheca is in a separate vial in the jar and, apart from being desiccated, complies with Lee’s (1959) account and figure. The wide intestine contains organic matter and is full of coarse grits of various minerals.

**Figure 5.**
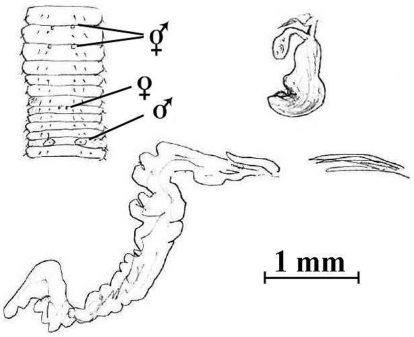
*Notoscolex tasmani* (Lee, 1959). Holotype (AMNZ 5039); sketch of anterior body (after Lee 1959: fig. 326), a desiccated spermatheca from vial (cf. Lee 1959: fig. 328) and flat, tubuloracemose prostate with penial setae *in situ* in 18lhs.

##### Remarks.

Having non-tubular prostates qualifies this taxon as a new combination in *Notoscolex*. The remote chance it acquires extra setae posteriorly after cut would permit it in *Anisochaeta*. Penial setae are unusual for New Zealand Megascolecidae, but it is interesting that they do not correspond well to the length of the spermathecal diverticula (see Blakemore 2000c; 2008). Further investigation is required for confirmation.

#### Aporodrilus equestris

(Benham, 1942) comb. n.

http://species-id.net/wiki/Aporodrilus_equestris

Fig. 6

Notoscolex equestris Benham, 1942: 220-225, Pl. 17, figs. 1–5; 1949: 348; 1950: 33; Lee, 1952: 37; Blakemore, 2004, 2006, 2010.
Megascolides equestris ; Lee, 1952b; 1959: 287, fig. 308 (of a spermatheca).

##### Distribution.

Poor Knights Islands, New Zealand [an online report, along with several other New Zealand earthworms, as a Marine invertebrate from Mexico - http://mexinverts.lifedesks.org/pages/1545 (Oct. 2011) is clearly a mistake].

##### Description from types.

Two specimens in jar: AMNZ 5040 a larger ~200mm specimen dissected previously, and AMNZ 5280 a smaller complete mature 140 mm long. Labelled “*TYPES Notoscolex equestris Benham 1942*”; “*Notoscolex Equestris Benham 1942*”; “*TAWHITI RAHI ISLAND, POOR KNIGHTS ISLANDS 26 November 1940 G.A. Buddle, R.A. Wilson, E.G. Turbott*”.

There is some slight confusion with *Notoscolex equestris* Benham, 1942, in that Lee (1959) erroneously placed it in *Megascolides*, and Lee (1959: 296) said types were in Otago Museum (No. A.43.52 - two specimens in fair condition but confirmation from Otago museum unforthcoming), yet he also gives “4 specimens. (Auckland Museum Collection)”. There are indeed two specimens in the Auckland Museum (pers. obs.) viz.: AMNZ 5040 with labels as above. Benham (1942: 220 - see http://rsnz.natlib.govt.nz/volume/rsnz_72/rsnz_72_03_002070.pdf) actually stated that Mr R.G. Turbott of the Auckland Memorial Museum had sent him two phials, one from Chatham Island that contained four earthworms, and these other two larger specimens collected from Poor Knights Islands by Majors G.A. Buddle and R.A. Wilson.

**Figure 6.**
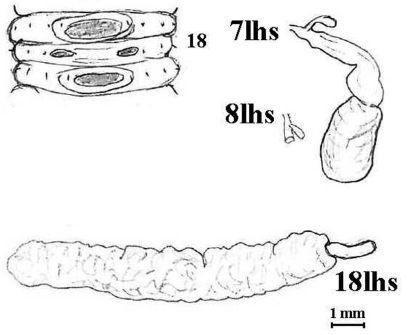
*Aporodrilus equestris* (Benham, 1942). Lectotype (AMNZ 5040); sketch of male field of paralectotype for comparison with Benham’s figures; also spermathecae (7lhs, 8lhs as a stub with missed diverticulum, 9lhs not shown) and prostate *in situ*.

Both specimens are here inspected and described: the larger one – that entirely agrees superficially with Benham’s figures – had been previously dissected with the 8lhs spermatheca, 18rhs prostate, and the anterior of the intestine removed and missing from the jar. Additions to Benham’s and Lee’s earlier descriptions are that the highly wrinkled prostomium is construed as pro-epilobous rather than prolobous, and no ventral cleft is present on the peristomium. Benham was “unable to detect the dorsal pores owing to the strongly contracted state of the body” and, for some reason, Lee omitted mention of them entirely except for exotic Lumbricidae. They are here confirmed as being absent throughout the body in both specimens (i.e., qualifying for *Aporodrilus*). Setae c and d are increasingly irregular. Spermathecae are in 7–9 but for 8lhs only the stub remains with the small diverticulum still attached (hence overlooked by earlier workers who also mistook slight folds in the soft duct as “excrescences”); as for other spermathecae, the small diverticula are visible by their slight iridescence just above the body wall at the base of the duct [see Fig. 6 and cf. Benham (1942: fig. 5), Lee (1959: fig. 308)]. Only the prostate 18lhs remains and is here construed as cylindrical tubulo-racemose i.e. non-tubular [see Fig. 6 and cf. Benham (1942: fig. 4)]. Genital markings agree as per original (Benham 1942: fig. 3) and the smaller undissected specimen (AMNZ 5280) is provided with a rough sketch showing how it conforms too. The gizzard appears more in 5 than 6 and oesophageal dilations are increasingly large in 10–14 (at least) but, as gut is removed, the intestinal origin cannot be confirmed. Although a typhlosole is absent and it is noted that the intestine below the break is filled with particularly coarse plant fragments only (no soil).

##### Remarks.

Both specimens are surely syntypes (one dissected agrees and key organs removed suggests they were figured by Benham, although Lee also dissected a prostate) and, under ICZN (1999: Art. 74) I hereby expressly designate the larger dissected specimen AMNZ 5040 the lectotype of *Aporodrilus equestris* (Benham, 1942) leaving the remaining undissected specimen as paralectotype (AMNZ 5080). In compliance with “Declaration 44 – Amendment of Article 74.7.3 of ICZN” (1999 – see http://iczn.org/content/declaration-44-amendment-article-7473), this act is in order to provide stability in its taxonomic name coupled with the augmented description provided herein. Enquiries made to verify Otago Museum material (Email: cody.fraser@otagomuseum.govt.nz 15^th^ Oct., 2011) were fruitless, but it is probable Lee (1959: 296) in his account confused the two lots that were sent to Benham, as commented on above.

### Note on genus *Aporodrilus*

**Figure 7.**
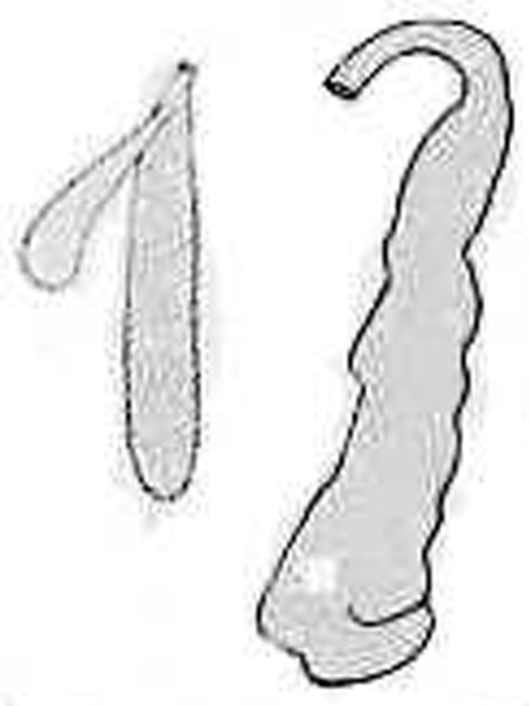
Spermatheca and prostate of *Aporodrilus mortenseni* from Michaelsen (1924: fig. 8).

Meroic *Aporodrilus* has lumbricine setae, tubuloracemose prostates and typically an intestinal origin in 16 (or 17) – as do many other species referable to *Notoscolex* – but it definitively lacks dorsal pores. Previously, only sixteen species and one sub-species were known, all from Tasmania (Blakemore 2000b, c; 2006). Lee’s (1952) species *Megascolides parvus* and *Megascolides viridis*, for example, also lack dorsal pores, but they differ in having tubular prostates; yet Lee’s statement that Michaelsen’s *Megascolex mortenseni* is in *Megascolides* seems slightly askew due to its lumbricine setae (at least in the anterior of the damaged specimen available to Michaelsen) and non-tubular prostates [see Michaelsen (1924: fig. 8b) – reproduced here as Figure 7] that would more properly place it in *Notoscolex*. Based on Michaelsen’s original description stating “Rückenporen sind nicht vorhanden”, it apparently now belongs in *Aporodrilus*. Since *Aporodrilus mortenseni* was only ever found in a garden at Palmerston North, it may be a translocated native *sensu* Blakemore (1999, 2008) rather than an exotic introduction as intimated by Lee (1959) who considered it outside the normal *Megascolides* range; its conservation status is currently unknown. With addition of *Aporodrilus equestris*, *Aporodrilus esculentus*, *Aporodrilus mortenseni* plus *Aporodrilus atoea* and *Aporodrilus ponga* spp. novae, the genus total increases to twenty-one species and its range extends from Tasmania to New Zealand (with other expected Australian members in Victoria and southern New South Wales). Relationships within this group remain to be determined.

### Note on oversights in Buckley et al. (2011) cladistic phylogeny

While inexplicably ignoring the Australian genus *Reflectodrilus* Blakemore, 2005, Buckley et al. (2011) stated:

*Australian Spenceriella were moved to Anisochaeta by Blakemore (2006), and they are in a weakly supported clade with Megascolex laingii, also transferred to Anisochaeta. The Anisochaeta concept has potential but needs revision based on more data. We provisionally reject the transfer (Blakemore 2006) of the Terriswalkerius species used here to Perionychella and/or to Diporochaeta because Terriswalkerius is a well-supported clade 5 nodes sister to the clade containing the other two genera*.

In actuality, Blakemore (2000c) was obliged – as any other taxonomist would be – to restore *Anisochaeta* Beddard, 1890 under ICZN priority as it had been overlooked and, rather than being a “concept” it is a valid and available prior genus with a tangible type-species, and hence acquires all similar species from *Spenceriella* Michaelsen, 1907 or any other synonymous genus. Genera strictly follow ICZN nomenclature and rather it is clades that are conceptual. Similar rules apply to genera *Diporochaeta* Beddard, 1890 and *Perionychella* Michaelsen, 1907 that take precedence over their successors for priority reasons as cogently explained by Blakemore (2000b, 2000c, 2005a). Thus, the phylogram by Buckley et al.(2011: fig. 2) merely confirms that all 15 species in the restricted group they call “a well-supported clade” should logically be folded into the earliest valid genus name which, in this particular case on a limited scope, they demonstrate to be *Didymogaster* Fletcher, 1887. The three proven NZ native families – Acanthodrilidae, Octochaetidae and Megascolecidae – may, by the same logic, be similarly telescoped back into family Ocnerodrilidae which is hardly a practical solution (see Blakemore 2005b).

Proper generic resolution hinges on molecular testing of *Diporochaeta* type-species, *Diporochaeta intermedia* (Beddard, 1888) from New Zealand. Whereas the four “*Diporochaeta*” samples these authors did test (SB 3 = *Diporochaeta chathamensis*?; WM5 an identical species they call “*Diporochaeta* n. sp. 1”; SB6 = *Diporochaeta brachysoma*, and an unidentified specimen from Tasmania) were partitioned into two separate “clades”, with (WM20) what they call “*Perionyx shoeanus*” or “*Perionychella shoeanus*” (sic) and “*Megascolides tasmani*”(WM83) intervening (cf. its treatment within). The only other *Perionychella* in their analysis, “*Perionychella kershawi*” (AF406567/ AY048484), is not only a misidentification, it is also the wrong species in the wrong genus as its proper title is “*Diporochaeta* cf. *kershawi*”. This last is certain as these specimens were personally collected, preserved and identified from Tasmania by the present author. Thus, rather than clarity we get further confusion and, as with several previous molecular phylogenetic works, the only errors in their otherwise informative study are the names.

Discussion of biogeography and phylogeny of NZ species is also somewhat invalidated by inability to differentiate genera when Buckley et al. (2011: 9) admit:

*The ﬁrst lineage (clade g) contains Megascolides and Spenceriella, the latter also labelled as Megascolides because they are intermingled with Megascolides proper*.

Since the last decade, *Spenceriella* Michaelsen, 1907 species have been subsumed in prior *Anisochaeta* Beddard, 1890, as noted above, and most NZ *Megascolides* McCoy, 1878 have now also been shown to belong in Australian *Notoscolex* Fletcher, 1887.

Finally, Buckley et al.’s argument for phyogenetic relationships of off-shore island taxa representing geological history or ocean currents largely ignores the human component whereby earthworms are frequently transported to new areas, inadvertently or sometimes deliberately, especially when used by Māori for fishing bait or food source (Benham 1905, Lee 1959: 304, Blakemore 1999, 2009) and as noted herein.

## Supplementary Material

XML Treatment for Aporodrilus aotea

XML Treatment for Aporodrilus ponga

XML Treatment for Notoscolex repanga

XML Treatment for Anisochaeta animae

XML Treatment for Notoscolex tasmani

XML Treatment for Aporodrilus equestris
